# Looking at the Road When Driving Around Bends: Influence of Vehicle Automation and Speed

**DOI:** 10.3389/fpsyg.2019.01699

**Published:** 2019-08-08

**Authors:** Damien Schnebelen, Otto Lappi, Callum Mole, Jami Pekkanen, Franck Mars

**Affiliations:** ^1^Laboratoire des Sciences du Numérique de Nantes (LS2N), CNRS, Nantes, France; ^2^Department of Digital Humanities, University of Helsinki, Helsinki, Finland; ^3^School of Psychology, University of Leeds, Leeds, United Kingdom

**Keywords:** gaze behavior, automated driving, look-ahead fixations, visuomotor coordination, steering control

## Abstract

When negotiating bends car drivers perform gaze polling: their gaze shifts between guiding fixations (GFs; gaze directed 1–2 s ahead) and look-ahead fixations (LAFs; longer time headway). How might this behavior change in autonomous vehicles where the need for constant active visual guidance is removed? In this driving simulator study, we analyzed this gaze behavior both when the driver was in charge of steering or when steering was delegated to automation, separately for bend approach (straight line) and the entry of the bend (turn), and at various speeds. The analysis of gaze distributions relative to bend sections and driving conditions indicate that visual anticipation (through LAFs) is most prominent before entering the bend. Passive driving increased the proportion of LAFs with a concomitant decrease of GFs, and increased the gaze polling frequency. Gaze polling frequency also increased at higher speeds, in particular during the bend approach when steering was not performed. LAFs encompassed a wide range of eccentricities. To account for this heterogeneity two sub-categories serving distinct information requirements are proposed: mid-eccentricity LAFs could be more useful for anticipatory planning of steering actions, and far-eccentricity LAFs for monitoring potential hazards. The results support the idea that gaze and steering coordination may be strongly impacted in autonomous vehicles.

## Introduction

Manual driving is a complex task that requires continuous processing of visual information to control the vehicle’s speed and lateral position, read traffic signs, examine potential hazards, etc. However, with the rapid development of autonomous vehicles research questions concerning the impact of automation on gaze strategies are increasing. In highly automated driving the driver delegates lateral and longitudinal control of the vehicle to the system. He or she becomes the supervisor of decisions made by the system. The driver is by definition outside the operational control loop, even without engagement in a secondary task, which can lead to changes in gaze strategies. The general objective of this study is to examine how gaze behavior differs between active (manual) and passive (automated) driving in curves, with a particular interest in anticipatory visual behavior under different spatiotemporal constraints.

The visual control of steering during active driving has been extensively studied and modeled. Commonly, steering is considered as depending on both feedback and feedforward processes (McRuer et al., 1977; for a recent review see Lappi and Mole, 2018). A compensatory feedback process may depend more on near-distance visual information (e.g., peripheral vision of the edge lines), and support corrections of lateral position (Summala et al., 1996; Mole et al., 2016). In parallel, an anticipatory feedforward process uses more distant visual information relating to changes in curvature of the road ahead. Precisely how gaze is functionally employed to supply visual preview information is a topic of ongoing research (Lappi and Mole, 2018). However, empirical observations from both on-road (Lehtonen et al., 2013, 2014; Lappi et al., 2017) and simulator studies (Wilkie et al., 2008; Mars and Navarro, 2012) suggests that the predominant gaze strategy during curve driving interleaves guiding fixations (GFs) with look-ahead fixations (LAFs).^1^

Guiding fixations are characterized by saccades to a point in the road 1–2 s ahead of the driver. These fixations provide a short-term preview of the road dedicated to the steering of the vehicle. GFs can be accommodated by a number of steering models in the theoretical literature (Lappi, 2014). Still, precisely how GFs provide preview, and the nature of visual information being sampled, has long been debated. Land and Lee (1994) hypothesized that the tangent point, a particular feature of the inner edge line, was functionally important during curve driving. Salvucci and Gray (2004) proposed an alternative model based on near and far points traveling ahead of the vehicle on the future path, although they acknowledged the driver can flexibly choose to look at the tangent point, a lead vehicle or any other relevant visual target depending on the driving situation (cf. also Lehtonen et al., 2018). On the other hand, Wann and Swapp (2000), Wilkie et al. (2010), and Lappi (2014) have argued that drivers look at waypoints on the future trajectory rather than at particular road characteristics. Additionally, Mars and Navarro (2012) proposed a view in which the driver is looking for the limits of an acceptable trajectory envelope (that often falls close to the tangent point). Although the debate on the exact nature of the GFs is still open, the common underlying assumption of these hypotheses is that the GFs promote visuomotor coordination for steering control.

Saccades to points in the road at considerably longer timescales than those commonly reported for GFs have also been observed. Under real driving conditions Kandil et al. (2010) reported that drivers spent one-third of the time looking at the bend exit as they approached the curve. Such fixations appear to be too far ahead to be useful for directly controlling the immediate steering trajectory. Further observations led Mars and Navarro (2012) and Lehtonen et al. (2013, 2014) to apply to curve driving the concept of LAFs, previously developed for manipulation tasks (Ballard et al., 1995; Land and Furneaux, 1997; Land et al., 1999; Pelz and Canosa, 2001; Hayhoe et al., 2003; Mennie et al., 2007; cf. also the gaze polling strategy observed by Wilkie et al. (2008) in a slalom task). LAFs in driving can be defined as intermittent glances directed further ahead on the road than where GFs are directed (i.e., significantly beyond the normal preview distance), with the gaze returning to the guiding preview distance.

Though the functional significance of each type of fixation is currently unknown, the overall gaze strategy has been described as a trade-off between the need to control the immediate steering trajectory (GFs), and the need to make longer-term navigation decisions (LAFs) (see Lappi and Mole, 2018, for a recent review). Such a trade-off would predict that reducing the need for steering corrections may “free-up” gaze to obtain anticipatory information from further ahead (i.e., make more LAFs).

Indeed, in a simulator study comparing automated and manual driving Mars and Navarro (2012) reported an increase in the proportion of LAFs and a concomitant decrease of GFs when the driver was in a passive driving condition (i.e., they did not need to move the steering wheel). It is possible that a higher proportion of LAFs improves the driver’s ability to anticipate, in line with Mackenzie and Harris (2015) who reported better hazard detection under passive driving conditions. However, an apparent increase in an ability to notice hazards (during automation as compared to manual control) does not seem to translate to an improved ability to safely respond to hazards: in Navarro et al. (2016) drivers that were passively driven showed more LAFs (than manual control) but poorer driving performance when an unexpected obstacle needed to be avoided. It is clear that being out of the operational loop causes disruption to steering and gaze control that cannot be compensated for by simply making more LAFs to get anticipatory information (for a detailed discussion see Mole et al., 2019).

In order to more fully understand the potential role of gaze during both manual and automated driving a better description of LAFs is required. The present study aimed to empirically define LAFs in curve driving. In particular, we set out to map the spatial and temporal characteristics of LAFs: how far, how long for, and how often (gaze eccentricity, fixation duration, and gaze polling frequency). The level of description provided will improve on previous research and allow researchers to investigate the influence of steering activity (or the lack of) on gaze by describing the shifting dynamics of GFs and LAFs. When manually driving, a tighter coordination between gaze and steering is required during corners rather than in the approaching straight line due to the need to compensate for changes in road curvature. Therefore, gaze distributions were analyzed depending on the section of the bends (approach or cornering). When passively driving, steering is performed by the vehicle so we might not observe large changes in gaze behavior between the approach or cornering phases. Therefore, it was hypothesized that differences in gaze behavior between active and passive driving would be larger in the Cornering section compared to the Approach section. Since gaze strategy is sensitive to the specific driving scenario the spatiotemporal constraints of the driving task were manipulated by modifying the speed of the vehicle. Anticipatory strategies (LAFs) were expected to change as a function of speed: when speed is high, the time available to perform LAFs and the time to reach to a potential hazard ahead is reduced, thus, shorter but maybe more frequent LAFs would be expected.

## Materials and Methods

### Experiment

#### Participants

Nineteen volunteers participated in this study. A full dataset was collected (i.e., there was no simulator sickness), though data from one participant have not been considered due to poor eye-tracker calibration. Mean age for the remaining participants (*N* = 18) was 26.1 years (SD = 4.2). Participants drove on average 9500 km per year (SD = 6500 km/year). To facilitate the recording of accurate gaze data participants had only normal or corrected (with contact lenses) vision. They also held a valid driving license and had no known neurological disease.

#### Equipment

**FIGURE 1:**
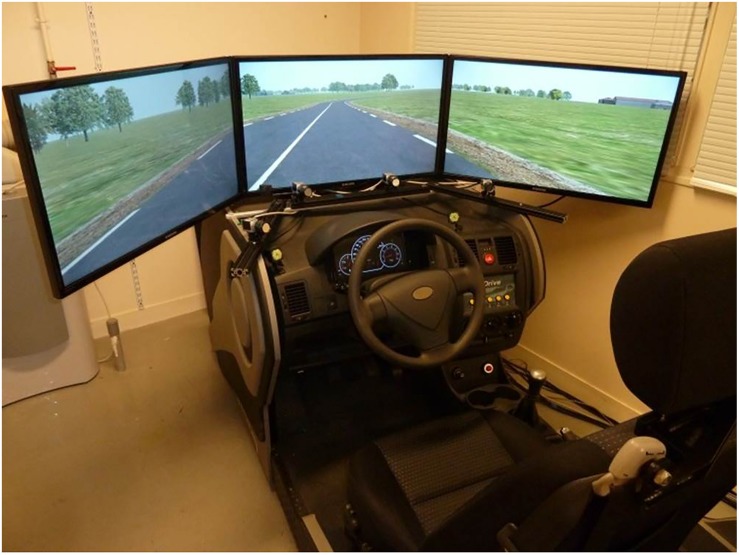
Driving simulator setup: the driving scene is displayed on three screens. The Eye Tracker was composed of four cameras.

The experiment was conducted using a fixed-base simulator that included an adjustable seat, a steering wheel with force feedback, a gear lever, clutch, accelerator and brake pedals, and a speedometer (see Figure 1). The driving simulator software was SCANeR Studio 1.4. The visual rendering of the road environment was displayed on three screens located in front of the driver, which offered drivers a 120° field of view.

Gaze data were recorded by means of a Smart Eye Pro (V5.9) eye-tracker with four cameras (two below the central screen and one below each peripheral screen). Calibration was achieved with 13 points, with the head fixed and oriented to the center of the central screen and the eyes directed to each calibration point in turn. Gaze position accuracy was given by the software: approximately 1.5° in the central screen, and 1.9° for more peripheral fixation points (standard deviation was 0.85° on average). Both eye-tracking data and vehicle position data were acquired at 20 Hz and synchronized by the driving simulator software.

#### Road

The test route was a 3 km two-lane rural road with no traffic. The width of the lane was 3.5 m for the whole track, with a continuous line in the middle of the road and broken markings for edge lines. LAFs are hypothesized to have a role in medium to long-term trajectory planning (Lappi and Mole, 2018), so it was considered that a track with curves of a variety of radii and lengths would elicit more LAFs than a track with predictable curves. However, since gaze strategy is heavily dependent on the unfolding track, curves with different parameters are effectively an uncontrolled confound when analyzing gaze data. Therefore, only six identical curves were retained for data analysis.

**FIGURE 2:**
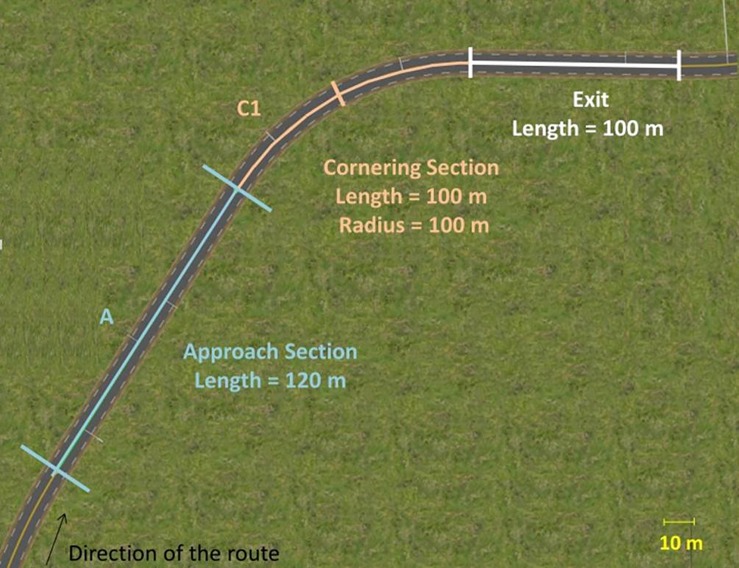
Geometry of the curves used for data analysis. The curves were composed of an Approach section (length = 120 m, straight line), a Cornering section (length = 100 m, radius = 100 m) and an exit section (length = 100 m, straight line). The analysis of gaze was performed in the first half of the Cornering section (C1) and in the Approach section (A).

To allow drivers enough time to anticipate as they approached the test bends (at least 4 s), a straight line of 120 m preceded the bends. This constituted the Approach section. For the Cornering section, the radius and length of the bend have been adjusted so that participants could pass through the bend in the three speed conditions (see Procedure). The bend radius was set at 100 m, and the arc length at 100 m. A 100 m straight line followed each bend. To make the road more easily discernible on the screen, this exit section was elevated of 1 m. Only the orientation differed between the bends: half of them were oriented to the left and three to the right (see Figure 2). The entire Approach section (A) and the first part of the turn section (C1; length = 50 m) were studied in the data analysis.

#### Procedure

First, the simulator seat position was adjusted to ensure that the participant was comfortably settled. Participants then drove manually along a training track to familiarize themselves with the driving simulator setup and the simulator environment. Since there is considerable heterogeneity in how familiar participants are with virtual environments the practice phase was open-ended and ended only when the participant felt comfortable with the vehicle and virtual environment. The practice phase lasted approximately 5 min.

After practice the eye-tracker calibration was performed and the participants were instructed that they would experience two driving modes, passive and active driving:

• In the active driving condition, drivers were in charge of the lateral control of the vehicle. Participants were asked to always remain in their own lane.
• In the passive driving condition, the lateral position of the car was managed by the driving simulator software. Drivers were told that the car was fully automated, although they might have to return to manual control. In that case, a red steering wheel symbol would appear on all screens. This event actually never happened. The aim was to ensure that drivers maintained their attention to the road scene.

In both conditions, the speed was fixed using a cruise control system at three different speeds: 60 km/h, 75 km/h or 90 km/h. The speedometer was hidden from the participant to avoid irrelevant glances at the dashboard. The road was designed so that the vehicle reached the given speed before entering in the first Approach section.

Each participant drove the test route six times, taking part in all conditions (3 speeds × 2 driving modes). The order of presentation of the conditions was counterbalanced using a diagram-balanced Latin Square.

### Data Analysis

Investigating the balance between GFs and LAFs requires a method to parse a gaze signal into either GFs or LAFs. The data analysis was composed of five different steps:

1. Computation of a GF reference from the horizontal gaze distribution.
2. Identification of two areas in the visual scene: GFs area of interest (GF AOI, computed from the gaze distribution analysis) and look-ahead fixations area of interest (LAF AOI).
3. Segmentation of eye-movements into fixations/pursuits and saccades.
4. Definition of gaze polling events based on eye-movements and gaze landing area.
5. Calculation of dependent variables and statistical inference.

The following subsections fully explain the processes taken in each analysis step.

#### Calculation of the Reference for Guiding Fixations

**FIGURE 3:**
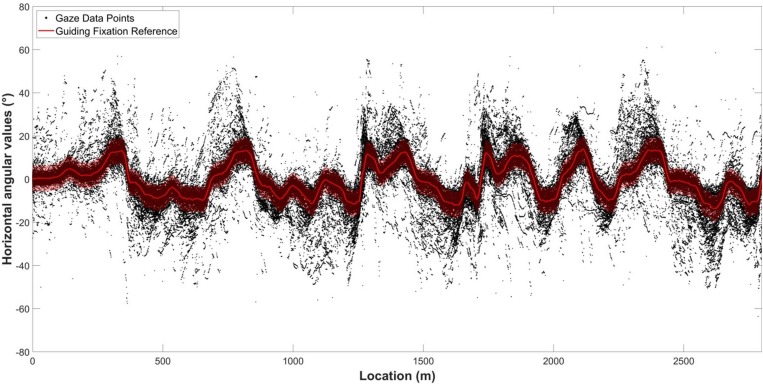
Visualization of the GF reference on raw data. The value of the GF reference was obtained using the filtered median horizontal angular value for all participants at each location along the track.

Gaze behavior in curve driving is fairly stereotypical, with the gaze following the road ahead 1–2 s ahead of the driver. This GF behavior describes the bulk of gaze observations, with the occasional LAF interspersed. There are two conceptual alternatives when identifying GFs. One is to define a priori some geometrical GF reference points, such as 2 s time headway on the future path (Lehtonen et al., 2014) or the tangent point (Land and Lee, 1994; see Lappi, 2014, for discussion). However, differences in gaze distribution relative to such points might be elicited by participants’ preferences, speed or other unknown variables. To take this into account, we opted for an alternative approach of deriving the GF reference position empirically from the observed gaze distribution (as used in Lehtonen et al., 2013) under the assumption that most gaze is GF. The median gaze eccentricity was used to determine the GF reference at each longitudinal position on the road. This analysis is accurate when the number of gaze measurements is high (so the influence of outliers is small), which was the case here (at least 108 measurements for each track position). In practical terms, gaze measurements of all participants in all conditions were collected for each position along the road and the median value was calculated for every 0.5 m interval. A third-order Savitzky–Golay filter with a 31 m window was applied to the median values in order to obtain a smoothed signal that defined the guiding reference, as illustrated by Figure 3. The following analyses were performed on horizontal deviations from that GF reference. The vertical deviations were not considered since they held very little information in our conditions (almost flat road). Indeed, the gaze angle varied mainly along the horizontal axis (SD = 20.3° for horizontal eccentricities vs. 3.1° for vertical eccentricities). In addition, although vertical deviations were used to distinguish fixations from saccades, it should be noted that gaze polling saccades are mostly horizontal (see section “Segmentation of Eye Movements”).

Two analyses were carried out separately, one for the bend approach, the other for the Cornering section.

#### Computation of a Guiding Area and Identification of an Anticipatory Area

**FIGURE 4:**
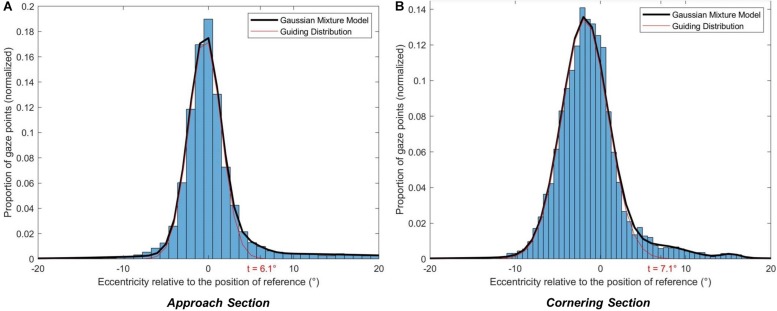
Threshold calculation. For each section (**A:** Approach, **B:** Cornering), the gaze distribution in the active driving condition was modeled with a Gaussian mixture model of 4 Gaussians. The main Gaussian (GF Gaussian) allows operationally defining the guiding fixations. The threshold (*t*) was set so that 99.95% of guiding fixations were below the threshold value.

To classify gaze observations as GF (or not), threshold values for the boundary of a GF AOI around the GF reference position is needed, such that it will encompass most or all of the GFs but few or no LAFs (or fixations in the scenery). In Lehtonen et al. (2013), a threshold value (6°) was chosen on the basis of visual inspection of data of a similar nature to Figure 3. A threshold approach by visual inspection is practical, but will be specific to the data, relies on the researcher’s prior assumptions of the functional role of LAFs, and is unlikely to translate well to different steering tasks (i.e., the thresholds may be overfitted to the data). Since the primary aim of the current study was to better describe gaze behavior in a way that is useful for researchers when analyzing gaze behavior on their own steering tasks, we set out to model the gaze distributions in a way that could be used to predict distributions for new steering tasks. Therefore, the GF AOI threshold value was determined on the basis of fitting a mixture of Gaussians to the gaze distribution obtained in the active driving condition. For the analyses, a positive eccentricity corresponded to a glance oriented toward the bend exit. With this sign convention, the gaze distribution is right-skewed due to anticipating glances (LAFs; see Figure 4). The rationale was to isolate one standard Gaussian distribution (with no skew) that encompasses GF, with the remaining gaze data points corresponding to anticipatory gazes.

Gaze distribution was modeled as a Gaussian mixture model: a weighted sum of components, each component following a Gaussian distribution. The minimum number of components was calculated to ensure the convergence of the model for gaze distributions in both the Approach and Cornering sections. The final number of components (*n* = 4) was selected using the Bayesian information criterion (BIC). Initial settings (Supplementary Table 1) and detailed results of the Gaussian mixture model can be found in the Supplementary Appendix.

Once the Gaussian mixture model was obtained for the Approach and the Cornering sections the central distribution (Gaussian 1 in Supplementary Tables 2, 3) was considered as the GF component. Then, the threshold for GF AOI was determined as gaze eccentricity values encompassing most (99.95%) of the GF component.

**FIGURE 5:**
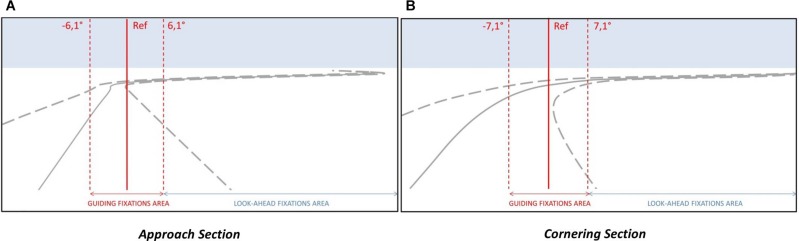
Schematic view of the driving scene during the Approach **(A)** and the Cornering **(B)** of the bend. The position of reference for guiding fixations as well as the threshold value allows defining two areas of interest: the guiding fixations area and the look-ahead fixations area.

Thresholds of 6.1° for the Approach section and 7.1° for the Cornering section were determined (see Figure 4). For each vehicle position gaze was classified as belonging to the GF AOI if the eccentricity (horizontal gaze angle) was below the threshold value. As positive eccentricities correspond to gaze oriented toward the bend exit, a LAF area could also be computed: if gaze eccentricity exceeds the threshold then it would belong to the LAF area. Figure 5 presents the obtained thresholds superimposed on schematic representations of the driving scene at two vehicle positions, one during the Approach and the other during Cornering. It can be observed at a qualitative level that the threshold matches up with the descriptions of GFs and LAFs in the literature: fixations that are directed ahead of the driver on the road, with a short time headway, are classified as GF; to be classified as a LAF the driver needs to look beyond the current trajectory and toward the bend exit.

#### Segmentation of Eye Movements

**FIGURE 6:**
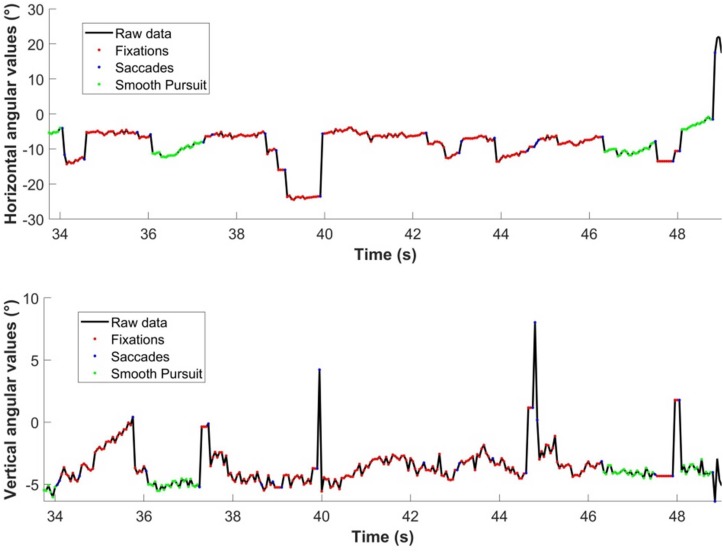
Segmentation of eye-movements using Naïve Segmented Linear Regression algorithm (Pekkanen and Lappi, 2017). Classification is obtained as a function of the shape of a given linear segment using Hidden Markov Models.

In order to quantify when drivers executed a saccade to a far (LAF) or nearer (GF) region it was necessary to parse the eye movement signal into saccades, and otherwise. The Naïve Segmented Linear Regression (NSLR) algorithm proposed by Pekkanen and Lappi (2017) appears to be an effective method of segmenting eye movement data into saccades, fixations and smooth pursuits. The segmentation treats angular gaze data and assumes that eye movements are reasonably approximated by linear segments. Then, for each new gaze data point, the algorithm calculates the maximum likelihood for this sample to belong to the prior linear segment or create a new one. The segmentation is performed by taking into account the slope of a given linear segment. An example of segmentation for one participant is presented on Figure 6.

#### Characterization of Gaze Polling

**FIGURE 7:**
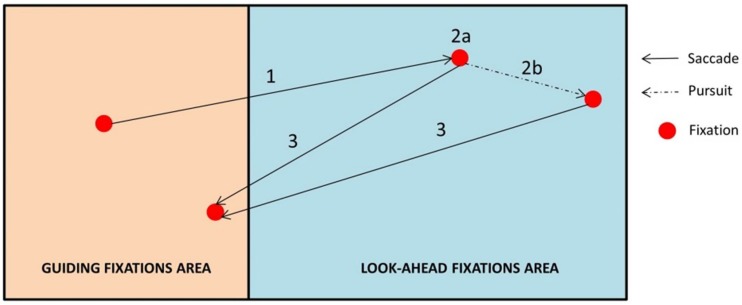
Characterization of gaze polling. The gaze shifts between guiding fixations and look-ahead fixation areas (1 → 2 → 3).

A gaze polling event (cf. Wilkie et al., 2008) was defined as the following sequence (Figure 7):

1. A saccade is launched from the GF area and lands in the LAF area.
2. Gaze remains in the LAF area for one or more fixations/pursuit movements.
3. The gaze returns to the GF area with a saccadic movement.

In this study we focused on the dynamics of shifts between GF and LAF areas (i.e., gaze polling events). For that reason, eye-movements occurring within the LAF area during gaze polling were treated as a single LAF, even though it may have included multiple saccades, fixations, or pursuits (see Figure 7).

In a given bend section (Approach or Cornering), a driver may look either within the GFs area only, either within the LAFs area only, or perform gaze polling. The number of bends with one of those visual behaviors was calculated depending on driving conditions.

When drivers performed gaze polling at least one time in a bend section, gaze polling frequency (total number of gaze polling events divided by duration of the given section), the mean LAFs duration, and the cumulative duration in percent (cumulative duration of all LAFs on a section divided by duration of the given section) were computed.

#### Dependent Variables and Statistical Analysis

##### Gaze distribution relative to the GF reference

The gaze distribution relative to the position of reference was computed between −11° and 18° using a 1° interval. Values outside the range (<−11° and >18°) were gathered in two extreme classes (total = 31 intervals). The values of −11° and 18° were chosen so that there was at least one data point per interval for each condition and each participant. All gaze data (fixations, saccades, smooth pursuits) were included in this analysis. Gaze distributions were analyzed in six conditions: speeds (60, 75 or 90 km/h) × driving condition (active or passive).

To allow comparison between left and right curves a sign convention had been used: a positive value of eccentricity correspond to a gaze directed toward the bend exit relatively to the position of reference. Differences between driving conditions at each bin were investigated with paired *t*-tests. Holm’s adjustment procedure (Holm, 1979) was used to control the type I error (α = 0.05) for the 31 (=number of bins) comparisons.

##### Look-ahead fixations during a gaze polling event

A 2 × 3 Type III ANOVA (2 driving conditions: active or passive, 3 speed conditions: 60, 75 or 90 km/h) with repeated measures was performed on gaze polling frequency, the mean duration of LAFs and the cumulative duration of LAFs during each section (Approach or Cornering). *Post hoc* analysis was performed using *t*-tests adjusted with Holm’s procedure for a level of significance at 0.05.

## Results

### Gaze Distributions

**FIGURE 8:**
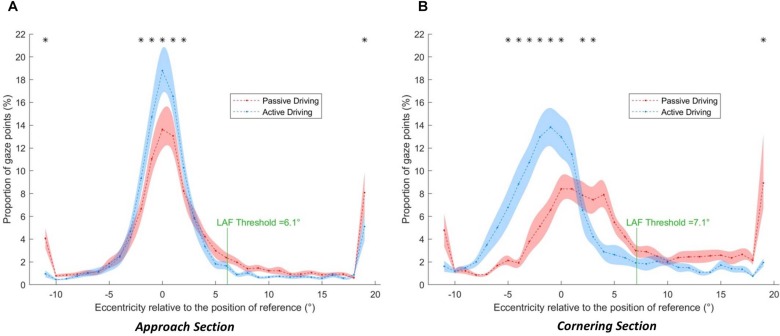
Gaze distributions as a function of the driving condition (blue: active, red: passive) and bend section (**A**: Approach, **B:** Cornering). The shaded area corresponds to the standard error of the means. Asterisks indicate a significant difference between active and passive driving at a given eccentricity.

Figure 8 shows the distribution of gaze as a function of driving activity in the Approach (a) and Cornering (b) sections. In all cases, the gaze distributions are right-skewed, with a non-zero mass above the LAF threshold and a large amount of data points in the positive extreme bin (any fixation above 18°). We shall henceforth describe fixations in the extreme category as far-eccentric LAFs, and fixations between the extreme category and the LAF threshold as mid-eccentric LAFs.

#### Approach Section

Descriptively, the peak of the both active and passive gaze distributions is located close to the GF reference (0°), within the interval [−1°, 0°], both in the active and passive driving conditions. The distributions are asymmetric with a higher proportion of gaze points directed toward the bend exit (positive values) than toward the opposite direction (negative values). One can notice that there is a larger proportion of gaze data points around the GF reference in the active driving condition than in the passive driving condition. However, passive driving gave rise to a higher proportion of gaze points that exceeds the LAF threshold (6.1° in the Approach section): 17.7% in the passive driving condition and 8.41% in the active driving condition (*t*(17) = 5.28, *p* < 0.001, *r*^2^ = 0.62). More precisely, far-eccentric gazes (directed toward the bend exit; extreme eccentricities >18°), accounted for 8.08% of all points in passive driving (5.10% in active driving). Mid-eccentric gaze points (from 6.1° to 18°) also represented 9.62% in the passive driving condition (3.31% during active driving).

Statistical analysis revealed a significantly higher proportion of gaze points in the active driving condition for eccentricities for all the intervals between −3° and 1°. Extreme eccentricities (<−11° and >18°) also reached statistical significance, with a larger proportion of far-eccentric LAFs made in the passive driving condition.

#### Cornering Section

In the Cornering section, the two gaze distributions are strikingly different. In the active driving condition, the peak of the distribution is located at the [−2°, −1°] interval, whereas in the passive driving condition the peak is wider with a large proportion of points between −1° and 4°. The total percent of gaze points that exceeds the LAF threshold (7.1° in the Cornering section) was 4.51% in the active driving condition and 32.3% in the passive driving condition (*t*(17) = 6.31, *p* < 0.001, *r*^2^ = 0.75). Moreover, gaze was rarely directed toward the far-eccentricities (>18°) (1.99%) or mid-eccentricities (2.52%) during active driving, whereas 8.42 and 23.78% of gaze points were directed there during passive driving.

Statistical analysis of the effect of driving condition on eccentricity revealed a significantly higher proportion of gaze points in the active driving condition for all eccentricities between −5° and 0° (*p* < 0.05 in all cases). On the other hand, a higher proportion of gaze points were found in the passive condition between 2° and 4° and for the far-eccentric LAFs (>18°).

#### Effect of Speed

**FIGURE 9:**
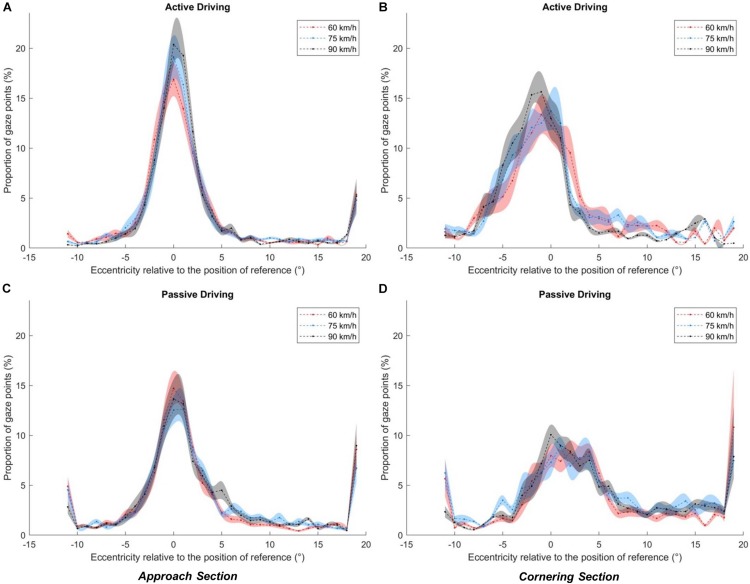
Gaze distributions as a function speed (red: 60 km/h, blue: 75 km/h, black: 90 km/h), bend section (**A,C**: Approach, **B,D**: Cornering) and driving conditions (**A,B** : active driving, **C,D**: passive driving). The shaded area corresponds to the standard error of the means.

There was not a significant effect of speed in either the Approach section or in the Cornering section (see the similar distributions across speeds in Figure 9).

### Detected Gaze Polling Events

**FIGURE 10:**
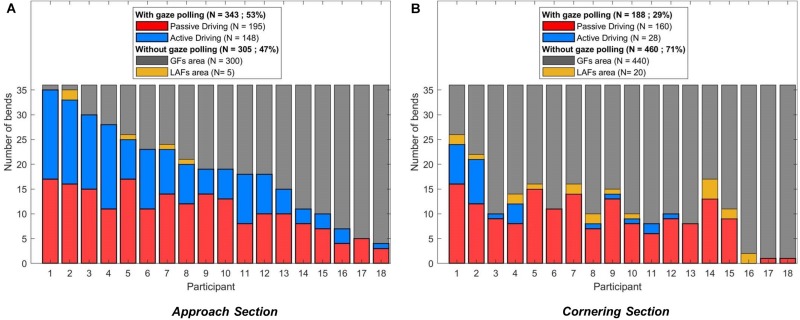
Gaze strategies adopted per participant in the Approach **(A)** and Cornering **(B)** section. Each bend was categorized as one of the four following behaviors: gaze polling during active driving (blue), gaze polling during passive driving (red), no gaze polling with the gaze always in the GF area (gray), no gaze polling with the gaze always in the LAFs areas (yellow). The participants are ordered according to their propensity to perform gaze polling in the Approach section.

Figure 10 summarizes in how many bends and in which driving condition drivers performed gaze polling.

Over a total of 648 bends (18 participants × 6 negotiated bends × 2 driving conditions × 3 speeds), drivers performed gaze polling in 343 bends (53% of all bends) in the Approach section. Gaze polling happened slightly more during passive driving (195 bends) than during active driving (148 bends). In the Cornering section, gaze polling was observed in 188 bends, with a larger difference between passive driving (160 bends) and active driving (28 bends) than in the Approach section. In other words, during active driving most drivers performed gaze polling only in the Approach section, whereas during passive driving most drivers performed gaze polling in both Approach and Cornering sections. In bends where gaze polling occurred, it represented 19.46% of all gaze data in the Approach section and 38.27% in the Cornering section.

In both sections, when no gaze polling was detected, the driver’s gaze remained in the GF area in all but 5 (Approach) and 20 (Cornering) bends. These exceptions, for which drivers spent all the time looking beyond the LAF threshold, always happened in the passive driving condition.

### Temporal Characteristics of LAFs During Gaze Polling

**FIGURE 11:**
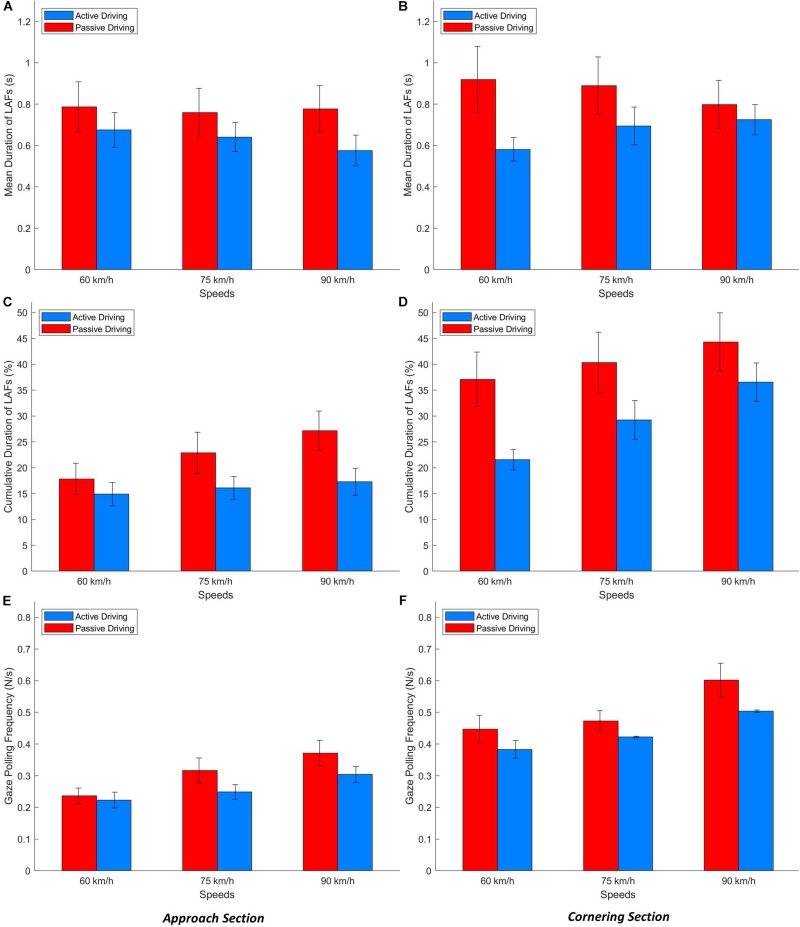
LAFs mean duration **(A,B)**, cumulative duration (in percent of total duration; **C,D**) and gaze polling frequency **(E,F)** as a function of speed and driving condition for the Approach **(A,C,E)** and Cornering **(B,D,F)** sections.

Figure 11 shows the gaze polling frequency, the mean duration of LAFs and the cumulative duration of all LAFs when gaze polling was performed.

#### Approach Section

The ANOVA revealed significantly higher values in the passive driving condition compared to the active driving condition for the mean LAF duration [*F*(1, 17) = 8.83, *p* < 0.01, $\eta_{p}^{2}$ = 0.023], the cumulative LAF duration [*F*(1,17) = 18.1191, *p* < 0.001, $\eta_{p}^{2}$ = 0.051], and the gaze polling frequency [*F*(1,17) = 9.81, *p* < 0.01, $\eta_{p}^{2}$ = 0.032].

The main effect of speed was significant only for the cumulative LAF duration [*F*(2,34) = 5.09, *p* < 0.01, $\eta_{p}^{2}$ = 0.036] and the gaze polling frequency [*F*(2,34) = 16.86, *p* < 0.001, $\eta_{p}^{2}$ = 0.091]: all values increased as a function of speed.

#### Cornering Section

For the Cornering section, higher values were found in the passive driving condition compared to the active driving conditions for all indicators. However, this difference was significant only for the cumulative duration [*F*(1,17) = 4.95, *p* < 0.01, $\eta_{p}^{2}$ = 0.051].

The effect of speed was significant only for the frequency [*F*(2,34) = 3.47, *p* < 0.05, $\eta_{p}^{2}$ = 0.12].

There was not a significant interaction between speeds and driving condition for both Approach and Cornering sections.

## Discussion

The current study examined gaze behavior for the Approach and Cornering sections of a bend, across active and passive driving conditions. During active driving a high concentration of gaze points was directed to the GF area with only few intermittent LAFs, mostly directed to the far distance toward the bend exit or beyond (cf. Lehtonen et al., 2014). This observation is in line with a number of on-road studies (e.g., Land and Lee, 1994; Lappi et al., 2013a, b, 2017; Lehtonen et al., 2013, 2014). The proportion of LAFs increased in passive driving, as was the case in Mars and Navarro (2012). However, in Mars and Navarro (2012) the bend was considered as a whole, whereas in the present study the LAFs made during the Approach and Cornering phase were considered separately. The distinction revealed that the increase in the proportion of LAF (or equivalently the decrease in the proportion of GFs) starts early, before entering the bend, but is much more pronounced when actually steering along the curve. When passively driving, LAFs represented more than 30% of all gaze data. By contrast, actively driving around corners gave rise to more than 95% of gaze points in the GF region below the LAF threshold (see Figure 8). The small amount of LAFs observed when actively steering around corners, and the proportionally larger amount of LAFs observed both before the bend and during passive driving, are consistent with the notion that GFs are involved in visuomotor coordination. When fewer steering corrections are required (so the need for visuomotor coordination is reduced), drivers preview the road further ahead, which gives rise to dramatically more LAFs.

If GFs are considered to be used for immediate lateral and longitudinal control of the vehicle, it follows that looking further ahead could provide anticipatory information over and above immediate trajectory control, for example upcoming maneuvers such as obstacle avoidance, gap acceptance, turning, and overtaking. While GFs guide ongoing action “just in time” (direct visuomotor coupling), LAFs could anticipate later actions (Mars and Navarro, 2012; Lehtonen et al., 2013, 2014; Navarro et al., 2016; Lappi and Mole, 2018). However, precise descriptions of GF function benefit from relatively tight time headway bounds and a long history of modeling the visual control of steering (Lappi, 2014). LAFs, on the other hand, encompass a much wider range of time headways so are a considerably more heterogeneous classification. For example, in the current study a glance is categorized as a LAF if it is more eccentric than the GF threshold but there is no upper limit to how eccentric a LAF can be, therefore LAFs could be just beyond the GF region or they could be very distant indeed (Figure 8). It would be unreasonable to assume that LAFs of such different eccentricities have identical functions.

Thus, we have made a distinction between LAFs of different eccentricities: mid-eccentricity and far-eccentricity LAFs. This distinction is particularly obvious when considering the results obtained in the passive driving condition during cornering (Figure 8). Here the gaze distribution was characterized by a number of far-eccentric LAFs that was twice as large as in the approach phase, but also by a large number of mid-eccentric glances directed between the GF area and the far-eccentricity LAF area. This suggests that for advanced information drivers did not always look “as far as they could see,” but also sampled the road at an intermediate distance.

This distinction between mid-eccentricity and far-eccentricity LAFs has never been really discussed before. In Mars and Navarro (2012), the driving activity only influenced the balance between GFs and far-eccentric fixations. As a consequence, the definition of LAFs was restricted to far-eccentric fixations. LAFs were considered as anticipatory glances to assess future road features (e.g., is the upcoming bend followed by a straight section or by another bend which may be hard to negotiate?) or traffic hazards (e.g., is there oncoming traffic or an obstacle on the road?). In other words, LAFs were thought as serving decision making, such as choosing to decelerate to anticipate a detected hazard or to accelerate when the road was assessed as safe to drive.

On the other hand, Lehtonen et al. (2013, 2014) used a single eccentricity threshold to separate GF and LAF, considering all fixations beyond GF as a single category. The authors reported mid-eccentricity LAF, similarly to what was observed in the present experiment. Those mid-eccentricity LAFs, mostly directed to the road, may not provide anticipatory information that is advanced enough to support decision making. Still, they are positioned too far to have a direct “online” contribution to steering responses. Lappi and Mole (2018) proposed that LAFs may rather be used for motor planning, allowing the selection and parameter setting of internal models of the vehicle-environment dynamics.

The results reported here illustrate this distinction: passive driving, by removing the need for direct visuomotor coupling leads to a much larger dispersion of gaze. Part of the gaze was dedicated to far eccentric features. We hypothesize that these LAFs mostly serve anticipation of future hazards (Mackenzie and Harris, 2015), although it cannot be excluded that a minor part of them was directed to parts of the visual field irrelevant to driving, such as looking at the scenery (the 2% increase of glances directed in the direction opposite to the bend may also reflect that). The rest of gaze behavior shows that drivers did not completely disengage from steering control. Indeed, mid-eccentricity LAFs suggest that drivers continued to gather information for trajectory planning in case a return to manual control was necessary, and there remained a substantial number of glances into the GF area.

Analyzing horizontal gaze distributions captures broad trends across conditions. However, one of the key reported characteristics of gaze behavior is the *interleaving* of GFs and LAFs. Therefore, individual bends were examined for the presence of specific gaze polling events (Figure 10). The results showed that gaze polling occurred more often during the Approach section than during cornering. Gaze polling was also more frequent in the passive driving condition (compared to active driving), this difference was especially pronounced when cornering. Interestingly, gaze polling did not happen in all bends. Moreover, drivers’ tendency to perform gaze polling showed large individual variations (similar to the results of Wilkie et al., 2008), suggesting that gaze polling may depend on participant’s preferences and driving experience.

During passive driving drivers performed gaze polling more often and also increased the time spent looking to the LAF area. Increased exploration of the visual scene during passive driving may lead to an improved situation awareness (Mackenzie and Harris, 2015), but it could equally mean partial disengagement from the driving task. In some rare cases (yellow data in Figure 10) the driver spent all the time looking at the LAF area. Looking far ahead may improve the ability to detect hazards well in advance. However, the absence of GFs may lead to poor visuomotor coordination and inadequate maneuver performance if these drivers were unexpectedly needed to take-over manual control (Navarro et al., 2016; Mole et al., 2019).

Finally, it is worth noting that while no effect of speed was found on gaze distributions gaze polling frequency did increase with speed, principally in the passive driving condition. This suggests that even if speed did not cause substantial alterations to the spatial distribution, it influenced the dynamics of gaze sampling. However, it should be noted that the range of speeds used in the study (60–90 km/h) was not very large, and as such would not substantially change the eccentricity of GF glances (cf. Tuhkanen et al., 2019). Larger differences in GF gaze distribution might be observed at very high speeds, which might in turn have an effect on gaze polling.

The current study shows differences in gaze strategy between active and passive driving over relatively short timescales. In future studies it will be interesting to examine how passive driving may affect gaze distributions and gaze polling dynamics over longer periods of time. A more prolonged period of automated driving may result in the driver being (further) out-of-the-loop (Merat et al., 2019), which can imply a degradation of perceptual-motor coordination (Mole et al., 2019) and a loss of situation awareness (Endsley, 1995). For instance, Körber et al. (2015) and Feldhütter et al. (2017) showed that drivers’ reaction time to events progressively increased with the duration of automated driving. It is possible that continuous assessment of gaze during automated driving could indicate when gaze has been redistributed and/or gaze polling dynamics have shifted, thereby providing clues as to whether a driver is safe to take-over control.

## Conclusion

The study analyzed gaze behavior when negotiating bends at various speeds when the driver was in charge of steering or when it was delegated to automation. When not driving gaze drivers looked further ahead, and polled more often between near and far regions. The distribution of gaze appears to support a distinction between two types of anticipatory LAFs: glances extremely far ahead (far-eccentric LAFs) that may be useful for hazard perception, and glances to an intermediary region (mid-eccentric LAFs) that may serve advanced movement planning. Speed did not change the global distribution of gaze but influenced gaze polling dynamics, in particular during the bend approach during automated driving. In the context of the current development of autonomous vehicles, in which the driver becomes a supervisor of the driving activity, this suggests that the monitoring of gaze behavior may serve the assessment of the driver state.

## Data Availability

The datasets generated for this study are available on request to the corresponding author.

## Ethics Statement

The experiment was approved by the INSERM ethics committee (IRB00003888 and FWA00005831). All participants gave written informed consent in accordance with the Declaration of Helsinki.

## Author Contributions

DS, OL, and FM designed the study. DS conducted the experiment. DS, JP, CM, and FM contributed to the data modeling and statistical analysis. DS, OL, CM, and FM interpreted the data and wrote the manuscript.

## Conflict of Interest Statement

The authors declare that the research was conducted in the absence of any commercial or financial relationships that could be construed as a potential conflict of interest.

## Footnotes

1 In the context of driving and more generally when an observer is in motion, the term “fixation” covers both oculomotor fixations proper and oculomotor pursuit of targets moving relative to the observer (gaze fixed on target, but eye not fixed in head; see Lappi, 2016). Moreover “GFs” and “LAFs” are used here as labels for gaze behavior that may include saccades that are large enough that in a laboratory study they would be considered to break fixation, that is, glance behavior. We use the terms GF and LAF to be consistent with the relevant literature, but use the term glance when discussing gaze data that has not been parsed into saccades, fixations and pursuits.
