# Supplementary material for: Looking at the Road When Driving Around Bends: Influence of Vehicle Automation and Speed
Source: Front Psychol. 2019 Aug 8;10:1699. doi: 10.3389/fpsyg.2019.01699 (PMC6694758; doi:10.3389/fpsyg.2019.01699)
Supplement: Supplementary file 1 [file Table_1.DOCX]

Supplementary Material – Appendix

## Initialization of the Gaussian Mixture Model

### Definition of a Gaussian Mixture Model

The model was set up using the function fitgmdist from Matlab. With this function, the Gaussian Mixture Model likelihood is optimized using an iterative Expectation-Maximization (EM) algorithm. This model fits a distribution as a sum of N components (each one following a Gaussian distribution) weighted by the proportion of each component. After convergence of the algorithm, gaze distribution (GD) is then modeled by:

$$GD= \sum_{i=1}^{N} C_{p_{i}}*N(\mu_{i} ; \sigma_{i})$$

where $C_{p_{i}}$is the proportion of component *i*, following a Gaussian distribution with mean $\mu_{i}$ and standard deviation $\sigma_{i}$.

### Initialial parameters and choice of the number of components

Gaussian Mixture models were estimated with several different initializations and the best fitting model was selected using the Bayesian Information Criterion (BIC). The 1st Gaussian Mixture Model comprised only a central component (Cp = 1; mean = 0; std = 5) to ostensibly capture the guiding fixations. For the two component model a component to capture the noise of the distribution (Cp = 0.1; mu = 0.0; std = 20) was added. For further models the initial parameters of the central (mean = 0.0; std = 5.0; and Cp = 0.5) and noise (mean = 0.0; std = 20 and Cp = 0.1) components remained the same and additional components were added with initial mean of 15, standard deviation of 2.0 and component proportion 0.4/(N-2) to capture glances directed toward the bend exit (positive eccentricities).


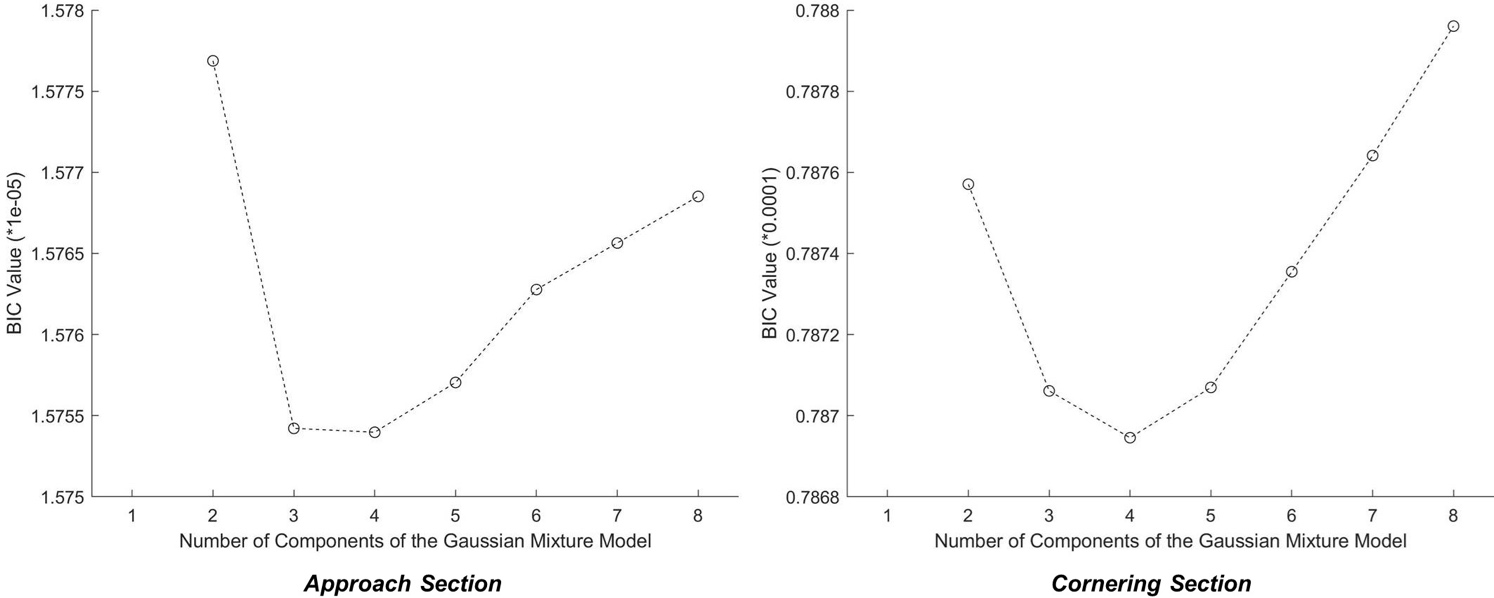


Figure 1: BIC values as a function of the number of components of the model for the Approach (a) and Cornering (b) sections. A blank remained for one component because the model did not converge in that condition.

The final number of components was decided by minimizing the BIC value for the two bend sections. The final number of components for both models was 4, with initial parameters given in table 1. The final estimated parameters are presented in sections 1.2 and 1.3.

| Properties | *Gaussian 1* | *Gaussian 2* | *Gaussian 3* | *Gaussian 4* |
| --- | --- | --- | --- | --- |
| *Data contained* | Guiding Fixations | Noise | Anticipating Glances | Anticipating Glances |
| *Mean (*𝛍*)* | 0 | 0 | 15 | 15 |
| *Standard Deviation*  *(𝛔)* | 5 | 20 | 2 | 2 |
| *Component Proportion*  *(Cp)* | 0.5 | 0.1 | 0.2 | 0.2 |

Table 1: Initial parameters of the Gaussian Mixture Model with 4 components.

## Gaussian Mixture Model of gaze distribution in the Approach section

After convergence, the parameters in table 2 were obtained in the Approach section.

| Properties | *Gaussian 1* | *Gaussian 2* | *Gaussian 3* | *Gaussian 4* |
| --- | --- | --- | --- | --- |
| *Data contained* | Guiding Fixations | Noise | Mid-term anticipation | Long-term anticipation |
| *Mean (*𝛍*)* | -0.4227 | 8.8337 | 5.2454 | 24.3850 |
| *Standard Deviation (𝛔)* | 1,9554 | 13.5282 | 1.4778 | 4.0340 |
| *Component Proportion (Cp)* | 0.8397 | 0.1326 | 0.0241 | 0.0035 |

Table 2: Final parameters of the model with 4 components in the Approach section

A visualization of each component of the model is presented in Figure 2.


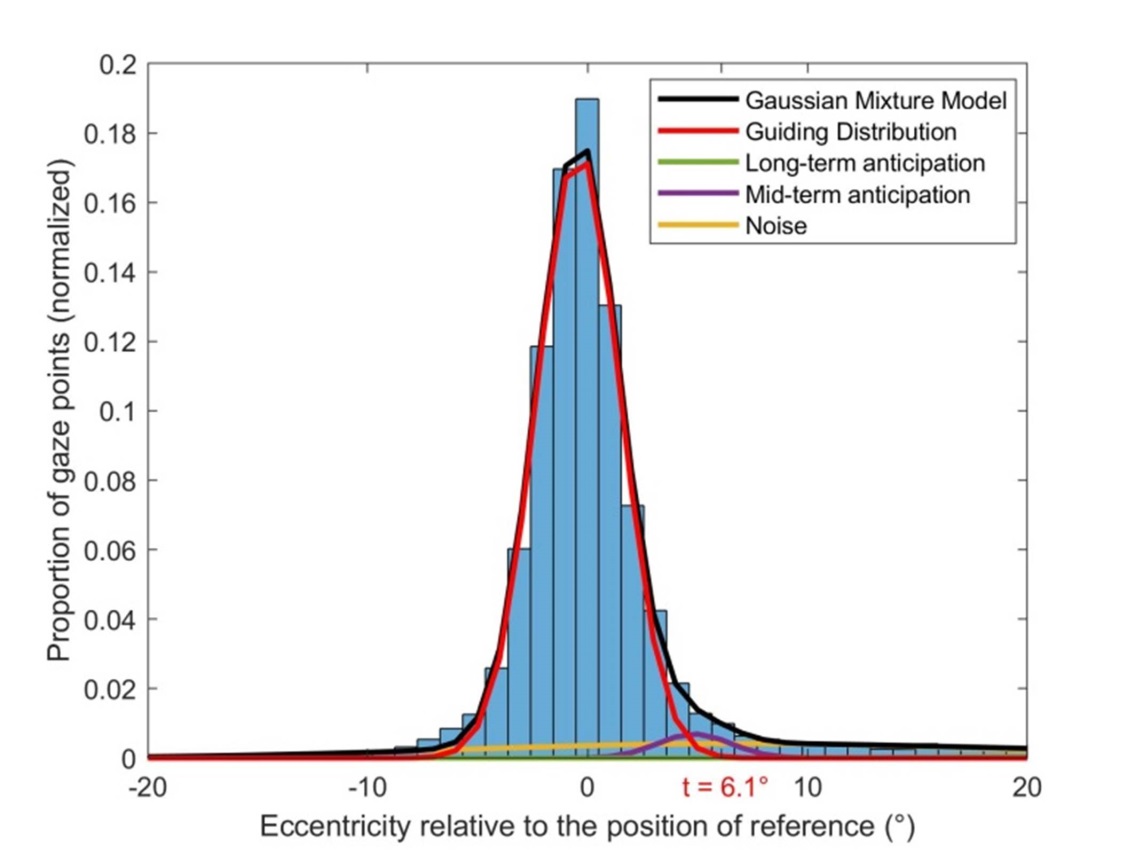


Figure 2: View of the Gaussian Mixture Model on the gaze distribution for the cornering section.

Each Gaussian represents one component of the Gaussian Mixture model.

## Gaussian Mixture Model of gaze distribution in the cornering section

After convergence, the parameters in table 3 were obtained in the cornering section.

| Properties | *Gaussian 1* | *Gaussian 2* | *Gaussian 3* | *Gaussian 4* |
| --- | --- | --- | --- | --- |
| *Data contained* | Guiding Fixations | Noise | Mid-term anticipation | Long-term anticipation |
| *Mean (*𝛍*)* | -1,8217 | 0.4857 | 7,2616 | 15,2994 |
| *Standard Deviation (𝛔)* | 2,6990 | 11,4790 | 2,9133 | 1,0237 |
| *Component Proportion (Cp)* | 0,9108 | 0.0401 | 0.0438 | 0.0055 |

Table 3: Final parameters of the model with 4 components in the cornering section

A visualization of each component of the model is presented on figure 3.


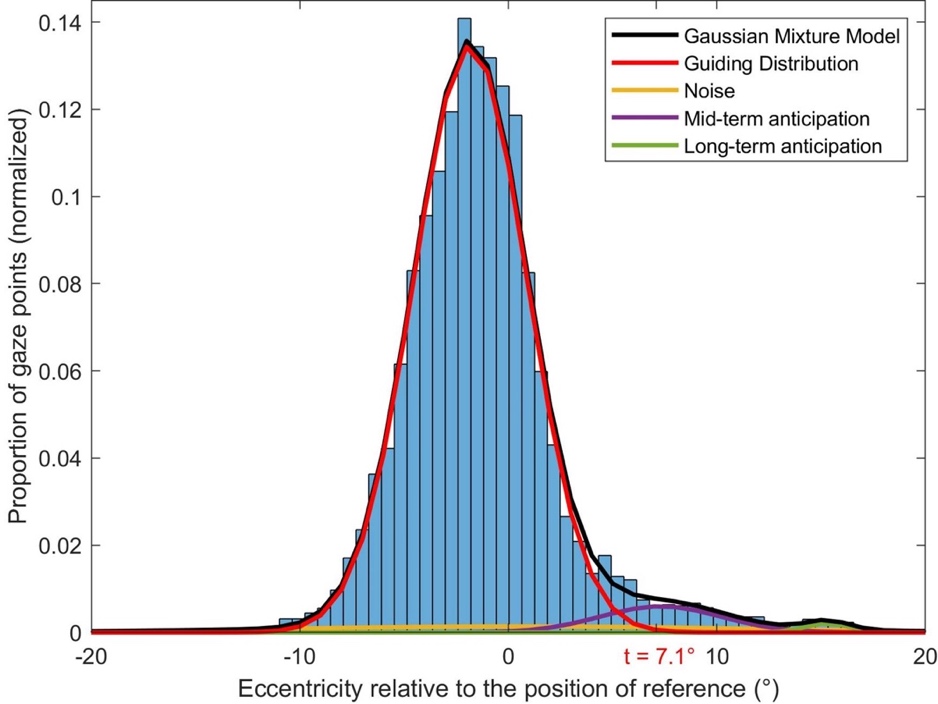


Figure 3: View of the Gaussian Mixture Model on the gaze distribution for the cornering section.

Each Gaussian represents one component of the Gaussian Mixture model.
